# 

**Published:** 1884-04

**Authors:** 


					﻿APRIL 1884
OLD SERIES
Vol. XXV
& 1855 O
NEW SERIES
Vol. I.—No. 2.

EDICAL-.cjURGIGAL
cdOURNAIi
v//t ■ St).

W.F.WESTMOREtAND,!®
H.V.M.MILLER,M.D.LL.D.>
JAMES A.GRAY, M.D?
PUBLISHED BY JAMES P.HARRISON&.CQ!
L	Atlanta,ga.
SUBSCRIPTION : 2.50 A YEAR.
				

